# Gene Mutation in Patients with Familial Hypercholesterolemia and Response to Alirocumab Treatment—A Single-Centre Analysis

**DOI:** 10.3390/jcm13185615

**Published:** 2024-09-22

**Authors:** Joanna Rogozik, Jakub Kosma Rokicki, Marcin Grabowski, Renata Główczyńska

**Affiliations:** 1 1st Department of Cardiology, Medical University of Warsaw, 02-097 Warsaw, Poland; jakub.rokicki@wum.edu.pl (J.K.R.);; 2 Department of Medical Informatics and Telemedicine, Medical University of Warsaw, 00-581 Warsaw, Poland

**Keywords:** alirocumab, APOB, familial hypercholesterolemia, genes, hyperlipidemia, LDLR, PCSK9

## Abstract

**Background**: Familial hypercholesterolemia (FH) is an autosomal dominant genetic disorder characterized by significantly elevated levels of low-density lipoprotein (LDL) cholesterol, which plays a major role in the progression of atherosclerosis and leads to a heightened risk of premature atherosclerotic cardiovascular disease. **Methods**: We have carried out an observational study on a group of 17 patients treated at the Outpatient Lipid Clinic from 2019 to 2024. **Result**: The most frequent mutation observed was found in the *LDL receptor (LDLR)* gene, which was identified in ten patients (58.8%). Five patients were identified to have a mutation in the *apolipoprotein B (APOB)* gene, whereas two patients had two points mutations, one in the *LDLR*, and the other in the *APOB* gene. The average age of patients with *LDLR* mutation was 54.8 (12.3); for *APOB* mutation it was 61.4 (9.3) and for patients with two points mutation it was 61.5 (14.8). The study results showed that at Week 12, individuals with *LDLR*-defective heterozygotes who were given alirocumab 150 mg every two weeks experienced a 63.0% reduction in LDL cholesterol levels. On the other hand, individuals with *APOB* heterozygotes experienced a 59% reduction in LDL cholesterol levels. However, in patients with double heterozygous for mutations in *LDLR* and *APOB* genes, there was a hyporesponsiveness to alirocumab, and the reduction in LDL-C was only by 23% in two individuals. **Conclusions**: In patients with a single mutation, there was a greater response to treatment with alirocumab in contrast to patients with double heterozygous mutation, who did not respond to treatment with PCSK9 inhibitors.

## 1. Introduction

Disorders involving lipids are pivotal in the progression of atherosclerosis and its associated clinical outcomes such as coronary heart disease and stroke. These disorders are a primary contributor to mortality in adult populations globally. However, timely identification and effective management can greatly diminish the likelihood of early cardiovascular events and enhance longevity. Familial hypercholesterolemia (FH), also referred to as primary hypercholesterolemia, is a genetic condition resulting from mutations that impair the ability to process and eliminate cholesterol. FH leads to extremely high levels of low-density lipoprotein (LDL) cholesterol, which is a key contributor to the development of atherosclerosis and raises the risk of an early onset of atherosclerotic cardiovascular disease (ASCVD) [1,2,3].

FH occurs in heterozygous mutation carriers (HeFH) and rare homozygous carriers (HoFH). HeFH in the general population has a relatively high prevalence of about 1 in 311 people [4,5], while HoFH is much rarer with a prevalence of about 1 in 1,000,000 [6]. Recent research indicates that the occurrence of HoFH is from 1 in 160,000 to 1 in 300,000 worldwide [5,7,8,9]. The estimated prevalence of FH in Poland is 1 in 250 individuals [10]. Genetically determined hypercholesterolemia is a medical condition that needs a proper and accurate diagnosis. Several diagnostic criteria for facilitating the diagnosis of FH have been established. These include the Dutch Lipid Network (DLCN), chosen most often in Poland to predict FH (presented in Table 1) [11], the 2017 Japan Atherosclerosis Society (JAS) FH criteria, the Make Early Diagnosis, a simplified Canadian definition of FH, the Prevent Early Deaths (MEDPED) criteria, the Simon Broome (SB) Register, and the Montreal-FH-Score (MFHS) [12,13,14,15,16,17,18,19]. These criteria help categorize FH as definite, probable, or possible. The diagnosis is established by identifying the characteristic physical symptoms, including the presence of arcus corneal, tendinous xanthomata, a premature history of cardiovascular diseases, and elevated LDL levels. Genetic testing is the gold standard for diagnosing FH due to its high accuracy [14,20,21]. However, it may not be practical for everyone due to its limited availability and cost. Clinical scores can also be utilized to diagnose FH, but may not be as accurate as genetic testing. Establishing an accurate diagnosis of FH is essential to ensure proper treatment and management of the condition, which can help prevent severe cardiovascular complications. The other reason to obtain a molecular diagnosis is to make it easier to conduct cascade screening for family members.

FH primarily arises from genetic mutations leading to the impaired function of the gene that encodes the *low-density lipoprotein receptor (LDLR*), *apolipoprotein B (APOB)* genes, or the increased function of the *proprotein convertase subtilisin/kexin type 9 (PCSK9*) gene [22,23,24,25,26]. These mutations are found in more than 90% of FH patients and are listed in Table 2. In autosomal recessive hypercholesterolemia (ARH) cases, biallelic variation in the *LDL-receptor adaptor protein 1 (LDLRAP1)* has been reported [27]. Genetic testing can detect changes in sequencing, deletion or duplication, in at least one CanGen in approximately 60–80% of individuals [28,29]. Although, it is worth noting that in about 20–40% of patients with the FH phenotype, these mutations may not be detected through genetic testing [29,30]. Polygenic hypercholesterolemia, which is caused by pathogenic mutations in unidentified genes or multiple genes, is another factor that contributes to FH [7,30].

Alirocumab is a monoclonal antibody that targets PCSK9, which is a protein that joins to LDLR found on the surface of liver cells and it competes with LDL for its binding to LDL receptors. When it occurs, it leads to the degradation of these receptors and is responsible for removing LDL-C from circulation. In addition, PCSK9 facilitates the breaking down of LDLR intracellularly for recycling. Alirocumab is a fully human immunoglobulin G1 antibody that effectively treats hypercholesterolemia in high-risk patients by blocking the binding of PCSK9 to LDLR. Through this process, it upregulates the number of receptors available for LDL-C removal, leading to a reduced concentration of LDL-C in the blood. Alirocumab can be employed as a standalone therapy or in conjunction with different lipid-lowering agents for patients who are unable to tolerate statins or for whom the administration of statins is medically prohibited.

Recent updates in European guidelines have revised the recommended target purpose of low-density lipoprotein cholesterol (LDL-C) for individuals who are at a significantly higher likelihood of experiencing major adverse cardiovascular events (MACE). Individuals who have been diagnosed with HeFH are classified as being at high to very high risk for developing cardiovascular diseases. For individuals with FH and atherosclerotic cardiovascular disease (ASCVD) who are at very high risk, the 2019 European Society of Cardiology/European Atherosclerosis Society guidelines suggest therapy aimed at achieving a minimum of 50% reduction from the initial values and LDL-C target of <1.4 mmol/L (55 mg/dL) [31]. If these goals cannot be met, a combination of drugs is recommended. In very high-risk FH patients who have not attained treatment goals with maximal tolerated statin plus ezetimibe, treatment with PCSK9 inhibitors (PCSK9-I) is recommended.

This retrospective study aims to present the genotype-specific effectiveness of alirocumab in treating individuals with heterozygous FH.

## 2. Materials and Methods

From 1 April 2019 to 1 February 2024, a comprehensive retrospective analysis was conducted at the University Clinic Center of the Medical University of Warsaw. This analysis focused on a cohort of 17 patients with FH who underwent our outpatient lipid clinic. Our study protocol was accepted by the Bioethics Committee at the Medical University of Warsaw (decision number AKBE/68/2023) and the ethics committee desisted the exigences for informed consent. The study was performed following the ethical guidelines of the 1975 Declaration of Helsinki. Our patients were identified through genetic testing. Molecular analysis was performed using the direct sequencing technique of the *LDLR* gene and a fragment of exon 26 of the *APOB* gene, as well as the MLPA technique. The material for the genetic test was DNA isolated from peripheral blood leukocytes. All genetic testing was completed before the patient’s referral to our outpatient clinic.

**Figure 1:**
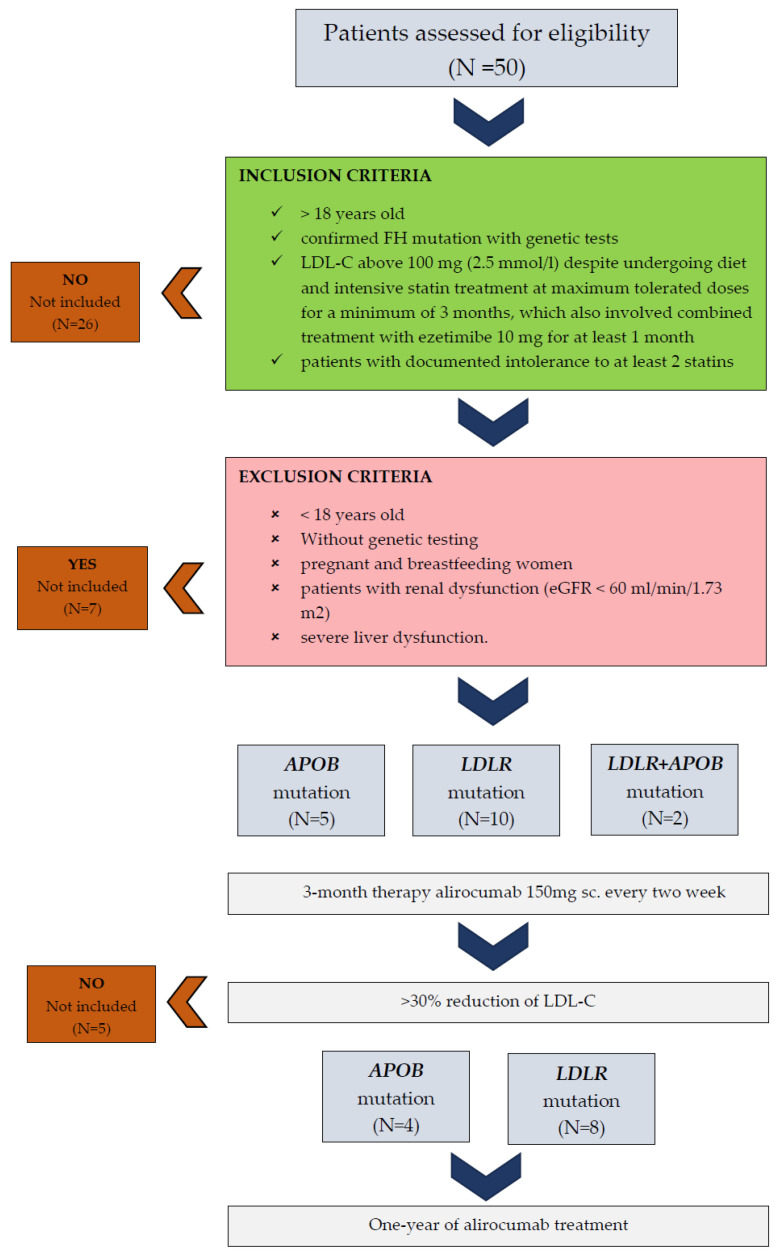
The stages of participant inclusion in the study.

Patients eligible for the study met the criteria of the Polish FH treatment program, including being over 18 years old, having a confirmed FH mutation with genetic tests, and maintaining an LDL-C above 100 mg (2.5 mmol/L) even when undergoing diet and intensive statin therapy at maximum tolerated doses for a minimum of 3 months, which also involved combined treatment with ezetimibe 10 mg for at least 1 month. Additionally, patients with a documented intolerance to at least 2 statins, as per the guidelines of scientific societies specializing in the diagnosis and treatment of lipid disorders, were also considered for the study. The exclusion criteria included individuals younger than 18 years, those who had not undergone genetic testing, pregnant and breastfeeding women, individuals with kidney impairment (eGFR < 60 mL/min/1.73 m^2^), and those with severe liver dysfunction. Figure 1 summarizes the stages of participant inclusion in the study.

The Dutch Lipid Clinic Network scale was employed to evaluate the patients. Alirocumab was applied subcutaneously at a dose of 150 mg once every two weeks throughout the treatment period. After the initial administration of the first two doses by a trained nurse in a clinical setting, patients were provided with the necessary training to self-administer four out of six doses of medication at home. The study involved measuring the lipid profile of individuals, including the concentration of total cholesterol (TC), LDL-C (calculated using Friedewald formula), HDL cholesterol (HDL), non-HDL cholesterol (non-HDL), and triglycerides (TG). The measurements were taken before treatment and 12 weeks after the start of treatment. To qualify for further therapy, patients must have achieved a reduction of at least 30% in serum LDL levels from the baseline. Participants who experienced a reduction in more than 30% in their baseline LDL-C levels were evaluated for ongoing alirocumab usage. The effects of alirocumab treatment after 1 year have been summarized separately. Additionally, alanine aminotransferase (ALT) concentration was measured to exclude patients with liver disease, and serum creatinine levels were assessed to evaluate kidney function as part of the initial qualification process. To evaluate the efficacy of treatment, we analyzed the impact of the detected mutation on the concentration of LDL-C before and after the onset of therapy.

### Statistical Analysis

Statistical analysis was performed using the MedCalc program. Group differences were presented as the mean value and standard deviation. Continuous variables were analyzed using nonparametric tests, specifically the Wilcoxon signed-rank test for paired samples and the Mann–Whitney U test for independent samples. The association of non-numeric variables was assessed using the chi-square test. Statistical significance was defined as a *p*-value < 0.05 for all tests.

## 3. Results

The study involved a sample of 17 individuals, with the majority being females (76.5%) and only 4 males. The mean age of the participants was 57.5 (11.1) years. The average age of patients with *LDLR* mutation was 54.8 (12.3); for the *APOB* mutation it was 61.4 (9.3), and for patients with two points mutation, it was 61.5 (14.8). Table 3 presents the baseline characteristics of the patients. The overall cholesterol level in the participants before initiating the therapy of alirocumab was 281.3 (85.1) mg/dL, while the LDL-C level was 206.1 (82.6) mg/dL. After 12 weeks of treatment with a dose of 150 mg of alirocumab every two weeks, the mean TC was 169.7 (98.9) mg/dL and LDL-C was 95.8 (95.7) mg/dL. In the study, 70% of patients were treated with combination therapy with the maximum tolerated dose of steroid and ezetimibe. Additionally, statin intolerance was identified in four patients. Among the patients, 35.3% were diagnosed with coronary artery disease, and two of them had a history of myocardial infarction. Carotid artery atherosclerosis was found in 29.4% of the patients, and three patients had a history of stroke.

The patients were evaluated concerning a confirmed mutation in the genetic testing of underlying FH. Among the enrolled population, the most frequent mutation observed was found in the *LDLR* gene, identified in ten patients (58.8%). Five patients had a mutation in the *APOB* gene while two patients were identified with mutations in both the *LDLR* and *APOB* genes. There were no patients with the *PCSK9* mutation in the study group. The genotype and correlation with phenotype are shown in Table 4. The most prevalent mutation observed among patients with *APOB* gene defects was mutation *p. R3527Q.* In the study, it was found that 60% of patients with the *LDLR* mutation displayed arcus cornealis before the age of 45, while two patients with a *p. R3527Q* mutation in the *APOB* gene exhibited tendinous xanthomata. The chi-square test did not show a significant association between the mutation and the occurrence of one of these phenotypes. No significant association was found between the concentration of either cholesterol fractions and the clinical presentation (tendinous xanthomata, arcus cornealis) typically associated with FH.

**Figure 2:**
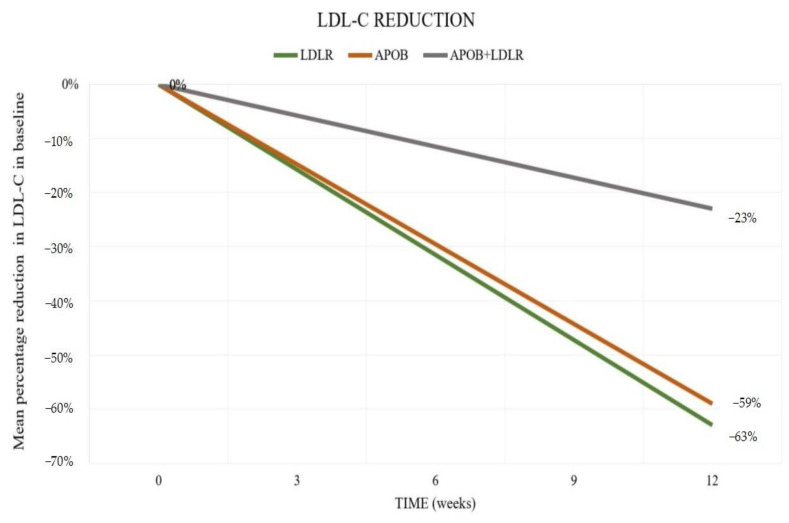
Mean percentage reduction from baseline in LDL-C level depending on the mutation after 12 weeks of treatment alirocumab in dose 150 mg sc. Abbreviations: *APOB*: apolipoprotein B gene; LDL-C: low-density lipoprotein cholesterol; *LDLR:* low-density lipoprotein receptor gene.

Patients demonstrated compliance with the prescribed treatment regimen and did not experience any adverse effects. The patients did not make any modifications to their diet or their previous lipid-lowering therapy. The study results showed that at Week 12, individuals with *LDLR*-defective heterozygotes who were given alirocumab 150 mg sc. every two weeks experienced a 63.0% reduction in LDL-C levels. On the other hand, individuals with *APOB* heterozygotes experienced a 59.0% reduction in LDL-C levels. Patients carrying single gene mutations, as previously mentioned, have been identified as responders to alirocumab therapy. However, in patients with complex *APOB + LDLR* mutations, there were non-responders to alirocumab, and the reduction in LDL-C was only 23% in two individuals (Figure 2).

**Figure 3:**
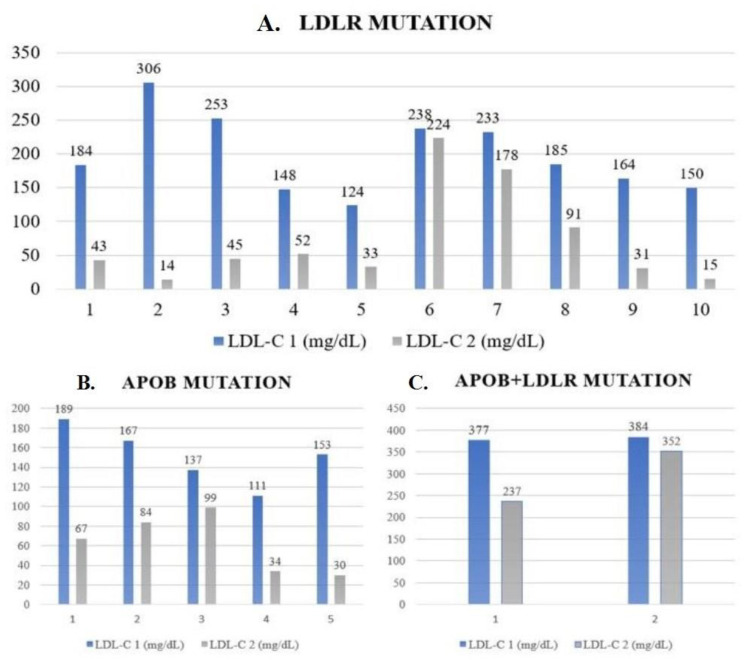
The LDL-C level fluctuations before and after a 12-week therapy of alirocumab for each patient. (**A**). Patients with *LDLR* mutation (n = 10). (**B**). Patients with *APOB* mutation (n = 5). (**C**). Patients with two genes mutation: *APOB + LDLR* (n = 2). Abbreviations *APOB:* Apolipoprotein B-100 gene; *LDLR*: Low-density lipoprotein receptor gene, LDL-C: lower-density lipoprotein concentration.

Figure 3 shows the LDL-C level fluctuations before and after a 12-week therapy of alirocumab for each patient. A single patient with a mutation in the *LDLR* gene showed a 95% reduction in LDL-C levels. This patient experienced a remarkable drop in LDL-C from a starting point of 306 mg/dL to 14 mg/dL after 12 weeks of treatment. However, one of the patients with combined *APOB+LDLR* mutation showed a poor response to treatment, with only an 8.3% decrease recorded from 384 mg/dL to 352 mg/dL.

### One-Year Follow-Up

Twelve patients who experienced a reduction in more than 30% in LDL-C levels from their initial values were evaluated for ongoing treatment with alirocumab. After 1 year of therapy, the mean LDL-C levels were 49.4 mg/dL (33.8 mg/dL) for the *LDLR* mutation group and 59.8 mg/dL (29.7 mg/dL) for the *APOB* mutation group. For the non-responder group, which comprised the remaining five patients, the average LDL-C level was 258.7 mg/dL (100.1 mg/dL). In patients with mutations in the *LDLR* gene, there was a notable decrease in LDL, non-HDL, and TC levels, indicating a positive response to treatment. However, this effect was not observed in HDL and TG levels. Conversely, no significant differences were found in the treatment effect among the group with *APOB* mutation, potentially due to the small sample size. In a 12-month observation, no significant variations in cholesterol fraction concentrations were noted between responders classified by identified gene mutations and non-responders grouped by gene mutations. One-year follow-up laboratory results are shown in Table 5.

MACE defined as a composite of nonfatal stroke, nonfatal myocardial infarction, and cardiovascular death was not observed during treatment with alirocumab.

## 4. Discussion

FH is a genetic condition caused by the *LDLR, APOB, PCSK9*, and *LDLRAP1* genes mutations. Recent studies suggest that the loss of function of the *LDLR* is responsible for a majority (60–90%) of all detected mutations [32]. Our research supports this finding, with patients possessing a confirmed *LDLR* mutation comprising 58.8% of our study population.

The most prevalent mutation observed among patients with *APOB* gene defects was mutation *p. R3527Q.* This single variant is found in >95% of patients population with FH cases caused by mutations in the *APOB* gene [33,34,35].

Alirocumab, a monoclonal antibody that targets protease PCSK9, has proven to be highly effective in patients with HeFH who require further reduction in LDL-C levels. The ALTERNATIVE study correlated alirocumab and ezetimibe in individuals at moderate to high cardiovascular risk with statin intolerance. The 12-week dosing of alirocumab resulted in a significant reduction in LDL-C by 47.0% (1.9) [36]. In a Phase 2 trial, treatment with alirocumab of 150 mg every 2 weeks resulted in an average LDL-C reduction of 67.90% (4.85) from baseline to week 12 [37]. The phase 3 clinical trials evaluated the percentage decrease in LDL-C levels after 24 weeks of drug administration. The latest research revealed that the mean LDL-C reductions from the baseline were as follows. In the FH I and FH II trials, individuals with HeFH and inadequate LDL-C control on maximally tolerated lipid-lowering therapy (LLT) were treated with alirocumab for 78 weeks. The LDL-C reductions observed were 48.8% and 48.7% in FH I and FH II, respectively [38]. In a LONG TERM study randomized controlled trial with high-risk patients with LDL-C > 70 mg/dL observed a significant reduction in LDL-C levels of 61.0% when alirocumab was added to statin therapy at the maximum tolerated dose. This treatment effect remained consistent over a 78-week period [39]. According to the ODYSSEY HIGH FH study, there was a 45.7% reduction in LDL-C levels among patients with HeFH and LDL-C levels of 160 mg/dL or higher, even though they were subject to the maximum tolerated statin therapy and/or additional lipid-lowering treatments [40,41]. Our research findings revealed that the mean percentage reduction in LDL-C among our study participants was consistent with the data reported for the population that received alirocumab treatment, as mentioned in the studies we referred to earlier.

The identification of mutation in genetic testing is important for understanding the efficacy of alirocumab treatment. Patients with a single mutation (*LDLR* or *APOB*) showed a more positive reaction to alirocumab treatment compared to those with double heterozygous mutation (*LDLR* and *APOB*), who did not have a response to PCSK9-I treatment.

The incidence of non-responsiveness to human PCSK9 monoclonal antibodies is very rare. Nevertheless, in such cases, clinicians need to consider the possibility of the presence of anti-drug antibodies, which can impact the efficacy of the treatment. During the ODDYSEY FH clinical trial, it was observed that 3 of the included 735 patients experienced positive alirocumab-neutralizing antibody status at one time, all at Week 12 [42]. The Phase 3 ODYSSEY studies investigated the phenomenon of apparent hyporesponsiveness to alirocumab, defined as less than a 15% reduction in LDL-C levels. This was observed in only 1% of the patient study population [42]. The possible reasons for this hyporesponsiveness could be a lack of adherence to therapy, a theoretical and infrequent opportunity of biological non-responsiveness caused by persistent antidrug antibodies, or other unidentified reasons.

Our patients showed a high level of tolerance towards alirocumab, and there were no reported adverse effects. According to the patient’s self-report, all prescribed doses were administered as scheduled following prior training. Nonadherence to the prescribed dosage regimen may have contributed to the observed lack of treatment response. However, the treatment was not effective, and we are still uncertain about the reason for this lack of effectiveness.

In the observation, during alirocumab treatment, there were no instances of MACE, which were defined as a combination of nonfatal stroke, nonfatal myocardial infarction, and cardiovascular death. In the ODDYSEY study alirocumab significantly reduced the risk of cardiovascular events [43,44].

The probability of finding double heterozygotes with two distinct genes is estimated to be approximately 1 in 1.4 million [45,46]. The combination of rare mutations in *LDLR* and *APOB* genes results in a more severe phenotype of atherosclerotic vascular diseases compared to either mutation alone. This is due to the cumulative effect of these mutations, which leads to elevated blood lipid levels [47]. Several studies have reported that individuals with double heterozygosity (mutations in both *LDLR* and *APOB* genes) may respond lower to PCSK9-I therapy [48,49]. Six studies found that individuals with primary mutations in genes linked to FH showed consistently positive responses to alirocumab therapy, with no significant differences observed [50]. The available evidence consistently showed that the LDL-C-lowering effect of PCSK9-I is consistent across genotypes [51]. The response to PCSK9-I was notably reduced in individuals carrying compound heterozygous and homozygous mutations across the published studies [51,52]. In the GENRE- FH study with evolocumab, human monoclonal immunoglobulin G2 that joins specifically to human PCSK9 had a similar lowering LDL-C effect comparable to alirocumab, whereas patients who did not have pathogenic variants or had only defective pathogenic variants experienced a higher percentage of achieved LDL-C reduction than those with at least one null pathogenic variant [53]. 

One of the primary limitations that ought to be considered when interpreting the results of the presented study is the small number of patients included in the analysis. Additional research is required using a more extensive sample of patients; we are planning on undertaking a cascade screening of patients’ families to investigate the underlying causes for the absence of noteworthy LDL cholesterol reduction in patients receiving alirocumab treatment. 

## 5. Conclusions

Patients with single mutation (*LDLR* or *APOB*) responded more to treatment with alirocumab than patients with double heterozygous mutation (*LDLR* and *APOB*), who did not respond to treatment with PCSK9-I. The administration of PCSK9-I was well-tolerated and did not lead to any severe side effects. No major adverse cardiovascular events occurred during the alirocumab treatment observation. Identifying double heterozygous through genetic testing is crucial for determining the suitability of alirocumab as a treatment for FH. This finding highlights the importance of genetic diagnosis in establishing a theoretical framework for personalized patient treatment.

## Figures and Tables

**Table 1:** Diagnosis of familial hypercholesterolemia based on Dutch Lipid Clinic Network criteria for FH [11] *.

Dutch Lipid Clinic Network Criteria	Points
(1) Family history	
First-degree relative with premature (men < 55 years; women < 60 years) coronary or vascular disease, or a first-degree relative with LDL-C above the 95th percentile	1
First-degree relative with tendinous xanthomata and/or arcus corneal, or children < 18 years with LDL-C above the 95th percentile	2
(2) Clinical history	
Patient with premature (men aged < 55 years; women < 60 years) CAD	2
Patient with premature (men aged < 55 years; women < 60 years) cerebral or peripheral vascular disease	1
(3) Physical examination	
Tendinous xanthomata	6
Arcus corneal < 45 years	4
(4) LDL-C levels (without treatment)	
LDL-C ≥ 8.5 mmol/L (≥325 mg/dL)	8
LDL-C 6.5–8.4 mmol/L (251–325 mg/dL)	5
LDL-C 5.0–6.4 mmol/L (191–250 mg/dL)	3
LDL-C 4.0–4.9 mmol/L (155–190 mg/dL)	1
(5) DNA analysis	
Functional mutation in the *LDLR, APOB*, or *PCSK9* genes	8

* A ‘definite’ FH diagnosis requires >8 points; A ‘probable’ FH diagnosis requires 6–8 points; A ‘possible’ FH diagnosis requires 3–5 points. Abbreviations: CAD—coronary artery disease; FH—familial hypercholesterolemia; LDL-C—low-density lipoprotein cholesterol; MI—myocardial infarction, PCSK9—proprotein convertase subtilisin/kexin type 9.

**Table 2:** The primary gene mutations found in FH.

Gene	Functions	Prevalence of Mutation	Mutation
*LDLR* *low-density lipoprotein receptor*	Uptake of low-density lipoprotein cholesterol (LDL-C), resulting in lower levels of LDL-C	60–90% of monogenic FH	Loss-of-function
*APOB* *apolipoprotein B-100*	Building of LDL-containing lipoproteins and transporting to the LDL receptor	5–10%	Loss-of-function
*PCSK9* *proprotein convertase subtilisin/kexin 9*	Recycling of LDL receptors is inhibited by promoting their demotion in the lysosomes	1–3%	Gain-of-function

**Table 3:** Baseline characteristics of patients.

Characteristic	*LDLR* Mutation (n = 10)	*APOB* Mutation (n = 5)	*APOB + LDLR* Mutation (n = 2)	All Patients (n = 17)
Age, year	54.8 (12.3)	61.4 (9.3)	61.5 (14.80)	57.5 (11.1)
Female sex- no (%)	7 (70.0)	4 (80.0)	2 (100.0)	13 (76.5)
Male sex- no (%)	3 (30.0)	1 (20.0)	0	4 (23.5)
DLCN	16.5	13.6	22.5	16.5
**Variables**				
Family history of ASCVD, n (%)	8 (80%)	5 (100%)	2 (100%)	15 (88.2%)
Hypertension, n (%)	1 (10%)	2 (40%)	0 (0%)	3 (17.6%)
T2DM, n (%)	1 (10%)	0 (0%)	0 (0%)	1 (5.8%)
Premature CVD	2 (20%)	0 (0%)	0 (0%)	2 (11.7%)
Myocardial infarction, n (%)	2 (20%)	1 (20%)	0 (0%)	3 (17.6%)
CAD, n (%)	3 (30%)	2 (40%)	1 (50%)	6 (35.3%)
PCI, n (%)	1 (10%)	0 (0%)	0 (0%)	1 (5.8%)
CABG, n (%)	1 (10%)	0 (0%)	0 (0%)	1 (5.8%)
Stroke/TIA, n (%)	1 (10%)	2 (40%)	0 (0%)	3 (17.6%)
Carotid disease, n (%)	5 (50%)	0 (0%)	0 (0%)	5 (29.4%)
PAD, n (%)	0 (0%)	0 (0%)	0 (0%)	0 (0%)
**Physical examination**				
Arcus corneal <45 y, n (%)	6 (60%)	1 (20%)	0 (0%)	7 (41.2%)
Tendinous xanthomata, n (%)	1 (10%)	1 (20%)	1 (50%)	3 (17.6%)
**Lipid-lowering treatment**				
Statins, n (%)	8 (80%)	5 (100%)	0 (0%)	13 (76.5%)
Ezetimibe, n (%)	9 (90%)	5 (100%)	0 (0%)	14 (82.4%)
Statin intolerance, n (%)	2 (20%)	0 (0%)	2 (100%)	4 (23.5%)
**Laboratory results before treatment**				
Total cholesterol (mg/dL)	272.8 (63.9)	228.4 (33.8)	456.0 (21.2)	281.3 (85.1)
LDL cholesterol (mg/dL)	198.5 (57.8)	151.4 (34.1)	381.0 (4.9)	206.1 (82.6)
HDL cholesterol (mg/dL)	50.1 (12.6)	59.4 (9.8)	56.6 (17.7)	53.6 (13.5)
non-HDL cholesterol (mg/dL)	223.6 (62.5)	169.0 (37.7)	399.5 (3.5)	228.2 (84.3)
Triglycerides (mg/dL)	130.8 (57.3)	88.8 (32.9)	95.0 (7.1)	114.2 (48.6)
Serum creatinine (mg/dL)	0.74 (0.1)	0.76 (0.1)	0.85 (0.1)	0.8 (0.2)
Estimated glomerular filtration rate (mL/min/1.73 m^2^)	97.3 (7.4)	88.0 (5.6)	76.0 (17.0)	92.1 (12.2)
ALT (U/L)	27.2 (13.5)	28.2 (12.2)	20.0 (5.7)	26.6 (12.0)
**Laboratory results after 12 weeks of treatment**				
Total cholesterol (mg/dL)	149.2 (81.2)	135.6 (46.4)	357.5 (89.8)	169.7 (98.9)
LDL cholesterol (mg/dL)	72.6 (73.1)	62.8 (27.9)	294.5 (81.3)	95.8 (95.7)
HDL cholesterol (mg/dL)	51.2 (16.5)	60.0 (23.1)	48.5 (9.2)	53.5 (17.1)
non-HDL cholesterol (mg/dL)	98.0 (68.4)	75.6 (29.0)	309.0 (80.6)	116.2 (93.4)
Triglycerides (mg/dL)	98.7 (39.4)	63.6 (28.1)	74.0 (4.2)	85.5 (36.4)

All data are presented as mean and standard deviation or as n (%). Abbreviations: ALT: alanine aminotransferase; ASCVD: atherosclerotic cardiovascular disease; *APOB:* apolipoprotein B gene; CABG: coronary artery bypass grafting; CAD: coronary artery disease; CVD: cardiovascular disease; DLCN: Dutch Lipid Clinic Network; HDL: high-density lipoprotein; LDL: lower-density lipoprotein; LDLR: lower-density lipoprotein receptor; non-HDL: non-high-density lipoprotein;T2DM: type 2 diabetes mellitus; PAD: peripheral artery disease; PCI: percutaneous coronary intervention; TIA: transient ischemic attack.

**Table 4:** Genotype and phenotype of each patient.

Patient (No.)	Age	Sex (F—Female M—Male)	Gene Mutation	Genotype	DLCN Points	Phenotype 1—Tendinous Xanthomata, 2—Arcus Corneal < 45y 0—None	CAD History (1—Yes, 0—No)
1	58	F	*LDLR*	*p.C95R, exon 3*	20	2	1
2	71	F	*LDLR*	*c.2311+56G>C/* *p.intron 15 c.941-2140del.*	21	0	0
3	40	F	*LDLR*	*c.314-1186dup, exons 4-8 duplication*	25	2	0
4	68	F	*LDLR*	*exons 4-7 duplication*	10	2	0
5	69	F	*LDLR*	*c.940+2T>C, exon 6*	15	2	1
6	55	F	*LDLR*	*p. G592E, exon 12*	12	0	0
7	57	M	*LDLR*	*c.1187-10G>A*	11	0	1
8	42	F	*LDLR*	*c.1775G>A p.* *R3527Q*	14	2	0
9	41	M	*LDLR*	*No data*	15	2	0
10	47	M	*LDLR*	*c.1871_1873del (p.I624del) exon 13*	22	1	0
11	51	F	*APOB*	*c.10580G>A/p.R3527Q exon 26*	12	0	0
12	60	F	*APOB*	*p.R3527Q exon 26*	10	0	0
13	66	F	*APOB*	*p.R3527Q exon 26*	15	1	1
14	73	F	*APOB*	*p.R3527Q exon 26*	19	2	1
15	57	M	*APOB*	*p.R3527Q exon 26*	12	0	1
16	51	F	*LDLR + APOB*	*c.1117G>T exon 8, c.10580G>A/p. R3527Q exon 26,*	25	1	0
17	72	F	*LDLR + APOB*	*p.G373C, exon 8, p.R3527Q, exon 26*	20	0	1

Abbreviations: *APOB*: apolipoprotein B-100 gene; CAD: coronary artery disease; DLCN: Dutch Lipid Clinic Network; *LDLR*: Low-density lipoprotein receptor gene.

**Table 5:** Laboratory results of patients after one year of treatment with alirocumab and patients showing no response to the treatment.

**Patients with Response to Alirocumab Treatment**				
Characteristic	***LDLR*** **Mutation (n = 8)**	***APOB*** **Mutation** **(n = 4)**	***APOB + LDLR*** **Mutation** **(n = 0)**	**All Patients** **(n = 12)**
Laboratory results after one-year treatment				
Total cholesterol (mg/dL)	126.5 (36.5)	133.3 (30.3)	0	128.8 (33.3)
LDL cholesterol (mg/dL)	49.4 (33.8)	59.8 (29.7)	0	52.8 (31.5)
HDL cholesterol (mg/dL)	51.8 (17.8)	57.3 (8.8)	0	53.6 (15.2)
non-HDL cholesterol (mg/dL)	74.8 (33.8)	76.0 (34.1)	0	75.2 (32.2)
Triglycerides (mg/dL)	111.1 (53.5)	76.3 (38.0)	0	99.5 (50.1)
**Patients with no Response to Alirocumab Treatment**				
**Characteristic**	***LDLR*** **Mutation (n = 2)**	***APOB*** **Mutation** **(n = 1)**	***APOB + LDLR*** **Mutation** **(n = 2)**	**All Patients** **(n = 5)**
Laboratory results after one-year treatment				
Total cholesterol (mg/dL)	378	209	407	344 (105.2)
LDL cholesterol (mg/dL)	288.5	127	331	258.7 (100.1)
HDL cholesterol (mg/dL)	69.5	72	51	65.5 (10.1)
non-HDL cholesterol (mg/dL)	308.5	137	356	277.5 (108.9)
Triglycerides (mg/dL)	99.0	52	125	93.7 (47.0)

Abbreviation: apolipoprotein B-100 gene; *LDLR*: low-density lipoprotein receptor gene, LDL: lower-density lipoprotein, HDL: high-density lipoprotein; non-HDL: non-high-density lipoprotein.

## Data Availability

The data presented in this study are available on request from the corresponding author, as they are not publicly available due to privacy concerns.
